# Anti-cancer Evaluation of Depsides Isolated from Indonesian Folious Lichens: *Physcia millegrana*, *Parmelia dilatata* and *Parmelia aurulenta*

**DOI:** 10.3390/biom10101420

**Published:** 2020-10-08

**Authors:** Ari Satia Nugraha, Tinton Agung Laksono, Lilla Nur Firli, Chintya Permata Zahky Sukrisno Putri, Dwi Koko Pratoko, Zulfikar Zulfikar, Ludmilla Fitri Untari, Hendris Wongso, Jacob M. Lambert, Carolyn T. Dillon, Paul A. Keller

**Affiliations:** 1 Drug Utilisation and Discovery Research Group, Faculty of Pharmacy, University of Jember, Jember 68121, Indonesia; tintona.l@gmail.com (T.A.L.); lillanurfirli13@gmail.com (L.N.F.); chintyapermata97@gmail.com (C.P.Z.S.P.); dwikoko.farmasi@unej.ac.id (D.K.P.); zulfikar.nazar@gmail.com (Z.Z.); 2 School of Biology, Faculty of Biology, Gadjah Mada University, Yogyakarta 55281, Indonesia; ludmilla.untari@ugm.ac.id; 3 School of Chemistry & Molecular Bioscience and Molecular Horizons, University of Wollongong, and Illawarra Health & Medical Research Institute, Wollongong, NSW 2522, Australia; hw765@uowmail.edu.au (H.W.); jl247@uowmail.edu.au (J.M.L.); carolynd@uow.edu.au (C.T.D.); 4 Labelled Compound and Radiometry Division, Center for Applied Nuclear Science and Technology, National Nuclear Energy Agency, Bandung 40132, Indonesia

**Keywords:** Indonesia, lichen, *Physcia millegrana*, *Parmelia dilatata*, *Parmelia aurulenta*, depsides, anti-cancer

## Abstract

Cancer is a serious health burden on global societies. The discovery and development of new anti-cancer therapies remains a challenging objective. Although it has been shown that lichen secondary metabolites may be potent sources for new anti-cancer agents, the Indonesian- grown folious lichens, *Physcia millegrana,* *Parmelia dilatata* and *Parmeila aurulenta,* have not yet been explored. In this study exhaustive preparative high-performance liquid chromatography was employed to isolate the lichen constituents with spectroscopic and spectrometric protocols identifying nine depsides **9**–**17**, including the new methyl 4-formyl-2,3-dihydroxy-6-methylbenzoate **13**. The cytotoxicity of the depsides towards cancer cells was assessed using the 3-(4,5-dimethylthiazol-2-yl)-2,5-diphenyltetrazolium bromide (MTT) assay. The results indicated lowest toxicity of the depsides towards human A549 lung cancer cells. Importantly, the di-depsides (**11**, **12** and **17**) showed greatest toxicity, indicating that these structures are biologically more active than the mono-depsides against the HepG2 liver cancer, A549 lung cancer and HL-60 leukemia cell lines.

## 1. Introduction

Lichens are unique organisms constituting a symbiosis between mycobiont and photobiont algae or cyanobacteria. The lichen structure comprises 50–90% of the mycobiont component leading to the taxonomical classification of lichen, being derived from its fungal component [1]. The global lichen population is comprised of 17,000–20,000 recorded species and accounts for 20% of total fungi metamorphosed into lichen. Some lichens are classified as extremophiles, as they can survive extreme climates including hot, cold, high humidity, high altitude and polar extremes. Secondary metabolite production can be related to adaptation processes, such as the synthesis of acidic compounds with disposition in cuticula providing protection from water diffusion during high humidity [2]. Hygroscopic metabolites were also secreted to directly absorb water from the environment [2].

Lichen secondary metabolites are produced mainly by the fungal components whereas primary metabolites are generated by the photosynthesis capable component [1]. The secretion of secondary metabolites by the mycobiont caused no damage to the photobiont component. Around 800 secondary metabolites have been reported from lichens in the form of depsides, depsidone, xanthone, anthraquinones, terpenes and fatty acid derivatives [3]. Although rare, lichens can also produce alkaloids and flavonoids [4]. Although lichen biomass is relatively less accessible compared to common medicinal plants, there are numerous examples as part of traditional medicaments to treat fever, diarrhoea, skin diseases and infection [5].

**Figure 1:**
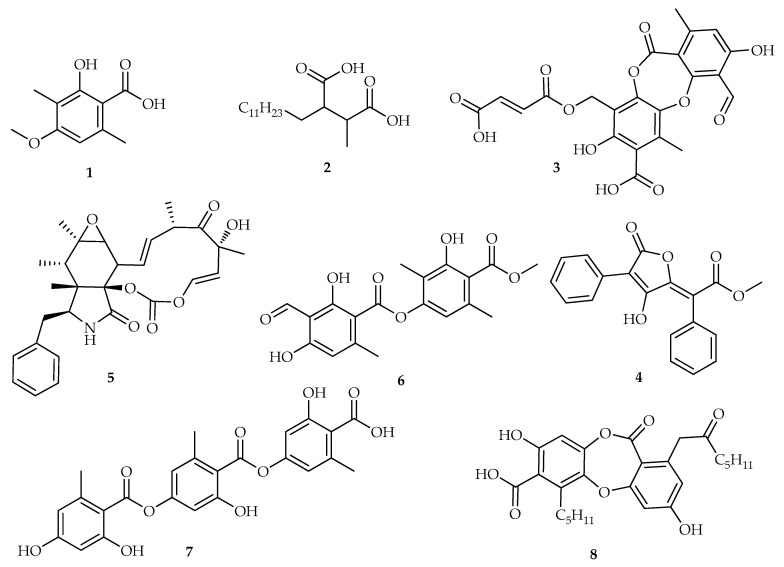
Notable anticancer active lichen secondary metabolites with succinic, lactam, depside, didepside, tridepside and a depsidone molecular backbone.

Previous notable reports on the anti-cancer activities of lichen secondary metabolites (Figure 1) include rhizironic acid **1** from *Parmelia erumpens*, which was toxic against A549, B16F10, and Caski cancer cell lines with IC_50_ values of 60.19, 2.53 and 2.69 µg/mL, respectively [6]. Isolated from *Roccella montagnei*, roccellic acid **2** showed significant anti-cancer activity against the DLD-1 colon cancer cell line with an IC_50_ value of 71.26 µg/mL. In silico evaluation led to the proposed mechanism of action being related to cyclin dependent kinase (CDK-10) inhibition, which is believed to prevent cancer cell proliferation [7]. Fumarprotocetraric acid **3** from *Cetraria islandia* and vulpinic acid **4** isolated from *Vulpicida canadensis* were active against MCF-7 cell lines with IC_50_ values of 19.51 and 58.02 µg/mL, respectively. These compounds also indicated neuroprotective activity on astrocyte cells [8]. The common fungal secondary metabolite, cytochalasin E **5**, was also reported as a lactam chemotype of *Pleurosticta acetabulum*,which possessed anti-cancer activity against the Widr cell line [9]. Other notable lichen secondary metabolites were atranorin **6**, gyrophoric **7** and physodic acid **8**, which showed in vitro anti-cancer potential against melanoma cell lines, with IC_50_ values ranging from 6.25–50 µM [10].

**Figure 2:**
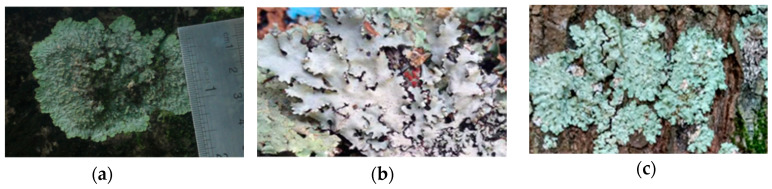
Folious lichen isolated from East Java-Java Island Indonesia: (**a**) *Physcia millegrana* Degel, (**b**) *Parmelia dilatata* Vain and (**c**) *Parmelia aurulenta* Tuck.

A previous notable study on lichens of Indonesian origin reported the cytotoxicity of the methanol crude extract from *Usnea misaminensis* against the KB cell, lung cancer cell NCI-H187 and breast cancer cell MCF-7 with IC_50_ values of 28, 11, and 30 μg/mL, respectively [11]. Lecanoric acid, a didepside from *Parmelia cetrata* was reported to be toxic against *Caenorhabditis elegans*, causing 80% mortality at a concentration of 100 μg/mL [12]. In a prior study on the *Physcia millegrana* L (Figure 2) crude methanol extract indicated anti-cancer activity against cancer cell lines, MCF-7, Widr, and Hela with IC_50_ values of 640, 332 and 137 μg/mL, respectively [13]. This study also investigated the *Parmelia* genus lichen, *Parmelia dilatata,* which revealed moderate anti-cancer activities for its crude methanol extract. The same result was reported from the preliminary study of *Parmelia aurulenta*. Although these species were common globally, there are no reports on their secondary metabolites as well as their bioactivities. In the current study, we report for the first time, the isolation of five mono and three di-depsides **9**–**12, 14**–**17** from these folious lichens. In addition, a new depside, methyl 4-formyl-2,3-dihydroxy-6-methylbenzoate **13**, was isolated. The cytotoxic (and subsequent) anti-cancer potential of these compounds was also investigated (using the 3-(4,5-dimethylthiazol-2-yl)-2,5-diphenyltetrazolium bromide (MTT) assay) and is reported in this study.

## 2. Materials and Methods

### 2.1. General

Analytical high performance liquid chromatography (HPLC) was performed using a Shimadzu LC-2030C-3D (Main unit) PDA with the Cooler HPLC system controlled by the LabSolutions LC Workstation Ver.5.73 Multi LC-PDA with a symmetry C_18_ RS column (5 µm, 4.6 × 150 mm). Preparative HPLC was performed on a Shimadzu Binary Preparative HPLC (LC-20AP Preparative HPLC Pump, CBM-20A, System Controller, SIL-10AP, Preparative Auto-sampler, SPD-M20A UV-VIS spectrophotometric detector) controlled by Lab Solutions LC Workstation Ver.5.3 with a Luna C_18_ RS column (5 μm, 19 × 150 mm) protected with a Security Guard PREP C_18_ (5 μm, 19 × 10 mm). Preparative HPLC was also performed using CECIL high performance liquid chromatography (HPLC) comprised of a CECIL CE4300 detector, a CE4040 degasser and a CE4104 pump, which were controlled by CE4900 and PowerStream software. A semi preparative column was used, YMC J’sphere ODS-M80 (10 × 250 mm 4 µm 80 Å). 1D-NMR spectra (^1^H and ^13^C NMR) and 2D-NMR spectra (gCOSY, gHSQC, gHMBC, zTOCSY) were recorded at 500 and 125 MHz, respectively, on a Bruker Avance 500 MHz. Absorbances for cytotoxicity assays were measured on a Molecular Devices BIORAD-BenchMark M550 microplate reader. Electrospray Ionisation Mass Spectrometry (ESIMS) spectra were obtained from a Shimadzu LC-2010 mass spectrometer in electrospray positive and negative ionization modes (ESI-MS). Preparative thin layer chromatography (TLC) was performed using Merck PLC silica gel 60 F_254_, 1 mm, 20 × 20 cm.

### 2.2. Sample Collection and Identification

Samples were collected on the University of Jember campus-Jember Regency and Klocing area-Bondowoso Regency, East Java, Indonesia on January–June 2018 with the voucher sample stored at the Faculty of Pharmacy, University of Jember under accession number B2, C5 and C4. Copies of the voucher samples were sent to lichenologist, Mrs. Ludmilla Fitri Untari, at the Faculty of Biology, Gadjah Mada University-Indonesia for identification.

### 2.3. Extraction, Separation and Isolation

A cleaned sample of *P. millegrana* (100.00 g) was ground into a powder with liquid nitrogen assistance followed by soaking with methanol (500 mL) and then stirred for 24 h. The supernatant was collected and vacuum dried. The process was repeated 3 times to produce the dried crude methanol extract (5.26 g). A portion of the extract (1 g) was mixed with methanol (10 mL) and was sonicated (30 min) and the resulting suspension was allowed to stand for one day, resulting in a precipitate which was then separated (white, 747.6 mg). The supernatant was filtered through an HPLC sample filter (0.45 µm) and subjected to preparative HPLC (19 × 150 mm, 5 µm) in 15 block injections. A gradient flow from 0–20% of solvent B within 2 min, 20–100% of solvent B within 20 min followed by isocratic development using 100% of solvent B for 5 min (solvent A: 100% water, solvent B; 100% acetonitrile) gave compound **9** (18.4 mg), **10** (3.4 mg) and **3** (15.0 mg) at *t*_R_ 14, 17 and 21 min, respectively. A portion of the precipitate (100 mg) was subjected to preparative TLC and elution with hexane:ethyl acetate (4:1) and produced compound **12** (24.7 mg).

For the *P. dilatate* sample, a clean lichen sample (55.74 g) was soaked in methanol (400 mL) and stirred for 24 h. The supernatant was separated and vacuum dried. The process was repeated 15 times to produce a dried crude methanol extract (9.55 g). A portion of the extract (50.1 mg) was mixed with methanol (1 mL) and sonicated (15 min). The supernatant was filtered through an HPLC sample filter (0.45 µm) and subjected to preparative HPLC (19 × 150 mm, 5 µm) in 11 block injections. A gradient flow from 0–40% of solvent B within 5 min, 40–100% of solvent B within 20 min, followed by isocratic development using 100% of solvent B for 5 min (solvent A: 100% water, solvent B; 100% acetonitrile) gave compounds **9** (4.2 mg) and **13** (4.4 mg) at *t*_R_ 13 and 17 min, respectively.

Methyl 4-formyl-2,3-dihydroxy-6-methylbenzoate **13**: UV active, brown amorphous solid (30.4 mg, mg.g^-1^ dry wt): IR (thin film, cm^-1^): 3296 (m), 2943 (m), 1645 (s), 1321 (s), 1263 (s), 1201 (s), 1172 (s); ^1^H-NMR ((CD_3_)_2_CO, 500 MHz): spectroscopic data see Table 1; ^13^C-NMR ((CD_3_)_2_CO, 125 MHz): spectroscopic data see Table 1; ESIMS-, *m*/*z* 209 [M – H]^-^. HRESIMS: calculated for C_10_H_9_O_5_ [M – H]^-^: 209.0450, found 209.0456.

For the *P. aurulenta,* a clean lichen sample (35.89 g) was soaked in methanol (400 mL) and stirred for 24 h. The supernatant was collected and vacuum dried. The process was repeated 15 times to produce a dried crude methanol extract (2.61 g). A sample of this extract (1 g) was redissolved in methanol (20 mL) and was sonicated (30 min) prior to filtration using an HPLC filter (0.45 um). The filtrate was subjected to preparative HPLC (1 mL pre injection). A gradient HPLC method was used from 0–20% of solvent B within 2 min followed by 20–80% of solvent B within 20 min. The development was continued with 80–100% of solvent B within 5 min. An isocratic development (100% solvent B) was employed for 3 min before the solvent was developed back to 100% solvent A within 2 min (solvent A: 0.1% trifluoroacetic acid (TFA) in distilled water, solvent B: 0.1% TFA in acetonitrile). This chromatographic protocol produced compounds **14** (2.5 mg), **9** (4.3 mg), **15** (11.2 mg), **16** (3.8 mg), **11** (4.5 mg), and **17** (25.1 mg) at retention times of 4.8, 10, 10.5, 20, 21 and 26 min, respectively.

### 2.4. Bioactivity Test: MTT Assays against Cancer Cell Lines

Iscove’s Modified Dulbecco’s Medium (IMDM), Dulbecco’s Modified Eagle Medium (DMEM), trypsin (2.5% *w*/*v* in phosphate buffered saline (PBS)), and penicillin/streptomycin (10,000 U/mL, 10,000 μg/mL) were purchased from Life Technologies (New York, NY, USA). Certified foetal bovine serum (FBS) was obtained from Bovogen (Waltham, MA, USA) and was heat-deactivated before use (60 °C, 1 h). CELLSTAR^®^ 96-well cell culture plates, and CELLSTAR^®^ filter cap cell culture flasks (75 cm^2^) were purchased from Greiner Bio-One (Kremsmünster, Austria). MTT (98%) and trypan blue (0.5% in PBS) were obtained from Sigma Aldrich (St Louis, MO, USA). PBS tablets (1 tablet per 100 mL of MilliQ water contains sodium chloride (0.8 g); potassium chloride (0.02 g); disodium hydrogen phosphate (0/115 g); potassium dihydrogen phosphate (0.02 g, Oxoid) and sodium dodecyl sulfate (SDS, > 99%)) were purchased from Thermo Fisher Scientific (Melbourne-Australia). Hydrochloric acid (HCl, 32%) was obtained from Ajax Finechem (Sydney-Australia).

The HL-60 cells used in these experiments were human acute promyelocytic leukemia cells derived from a 36-year-old Caucasian female with acute promyelocytic leukemia. The HL-60 cells were grown from semi-permanents (obtained from Associate Professor Ronald Sluyter (UOW); they were originally purchased from ATCC). The cells were grown in growth medium containing IMDM, FBS (20% *v*/*v*) and penicillin/streptomycin (200 units/mL and 200 μg/mL, respectively). The cells were grown in cell culture flasks, at 37 °C.

The HepG2 cells used in these experiments were human liver cancer cells derived from a 15-year-old Caucasian male with a well-differentiated hepatocellular carcinoma. The HepG2 cells were grown from semi-permanents (obtained from Professor Mark Wilson (UOW); they were originally purchased from ATCC). The cells were grown in growth medium containing DMEM, FBS (10% *v*/*v*) and penicillin/streptomycin (200 units/mL and 200 μg/mL, respectively). The cells were grown in cell culture flasks, at 37 °C. The HepG2 cells were harvested with trypsin (0.25% in PBS, 5 min incubation).

The A549 cells used in these experiments were human lung cancer cells derived from a 58-year-old Caucasian male with a lung carcinoma. The A549 cells were grown from semi-permanents (purchased from ATCC). The cells were grown in growth medium containing DMEM, FBS (10% *v*/*v*) and penicillin/streptomycin (200 units/mL and 200 μg/mL, respectively). The cells were grown in cell culture flasks, at 37 °C. The A549 cells were harvested with trypsin (0.25% in PBS, 5 min incubation).

The protocol for MTT cytotoxicity assays differed for the suspension versus the adherent cell lines, as described. The suspension cells (HL-60, 2 × 10^5^ cells/50 μL in IMDM) were added to the specified wells (rows 2–4, columns B–I) of a 96 well flat bottom plate and treated immediately. The HepG2 cells (1 × 10^4^ cells in 100 μL/well) and A549 cells (4 × 10^4^ cells in 100 μL/well) were seeded in growth medium and incubated for 24 h until the cells had adhered and were fibroblastic. At this point the growth medium was removed and fresh DMEM (50 μL/well) was added.

Stock solutions of each treatment solution were prepared (10,000 μg/mL, DMSO). The stock solutions were serially diluted in DMSO to achieve a range of concentrations that were 50 times more concentrated than the desired concentrations used for analysis. Each concentration of compound was then diluted by a factor of 1 in 50 with the treatment medium (HL-60, IMDM; HepG2 and A549, DMEM) immediately prior to treatment to provide the range of concentrations to be analysed (2% DMSO). The treatment solutions (50 μL) were added to each of the wells in columns C-I (most dilute in column C, most concentrated in column I), while IMDM or DMEM (50 μL) was added to the control well cells (column B). The plates were incubated (37 °C, 5% CO_2_) for 24 h, after which the MTT solution (20 μL, 5 mg/mL, PBS) was added to the wells and the plates were incubated (37 °C, 5% CO_2_) for 4 h to allow for formazan development. The solubilising solution (100 μL, 10% SDS in 0.01 M HCl) was added to the specified wells and the plates were incubated (37 °C, 5% CO_2_) overnight.

Absorbance readings were recorded at 570 and 690 nm (BMG LabTech Polarstar Omega microplate reader) and concentration–response curves (using Equation (1)) were produced in order to calculate the IC_50_ value for each compound.

(1) $$\mathrm{Cell}\;\mathrm{survival}\;\left(\%\right)=\;\frac{\left(A_{570}-A_{690}\right)\;\mathrm{treated}\;\mathrm{cells}}{\left(A_{570}-A_{690}\right)\;\mathrm{control}\;\mathrm{cells}}\;\times \;100$$

The calculated cell viability was plotted against the treatment concentrations using Microsoft Excel^TM^ [14] to determine the IC_50_ value of each compound. All MTT assays were performed at least three times to obtain reproducible IC_50_ values. Statistical analysis was performed with one-way ANOVA and Tukey’s multiple comparison test using GraphPad Prism [15].

## 3. Results

**Figure 3:**
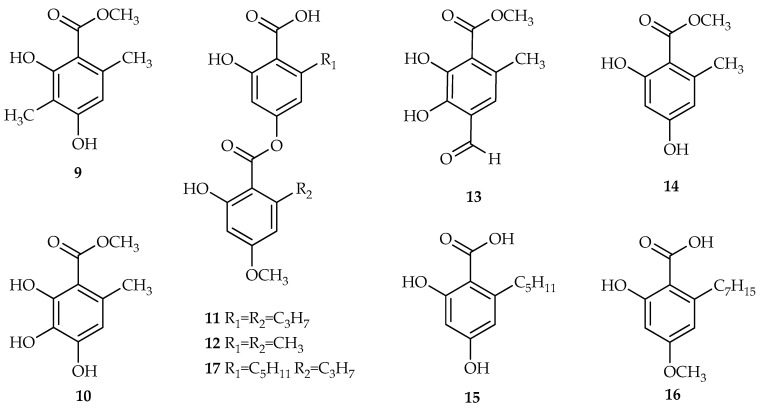
Mono and di-depsides isolated from *P. millegarana*, *P. dilatata* and *P aurulenta*.

The collection of lichen biomass is challenging due to its nature and the burden of ensuring that the samples were clean required extensive work. This is due to the small size of the folious lichens and the constraints on collecting samples—they were attached to the wood bark in which the folious was fully grown during wet season (January–June) leaving only the mid wet season as the best harvesting time. The crude methanol extracts were subjected to a short C_18_ cartridge (10 × 20 mm) for cleaning before separation by preparative high performance liquid chromatography (HPLC). The major component of the crude extract of *P. millegrana* was readily precipitated from methanol in which, by a simple preparative layer chromatography (PLC), the precipitate was repurified to produce evemic acid **12** as a white amorphous solid. This acid was previously reported from a different lichen family, *Umbilicariaceae* [16]. Preparative HPLC successfully isolated four compounds from *P. millegrana,* atraric acid **9** [17], methyl 3-hydroxy orsellinate **10** [18] and divaricatic acid **11** [19], and represents the first time these compounds have been isolated from the *P. millegrana* lichen species. The separation and isolation of the *P. dilatata* crude extract produced evemic acid **9** and a previously unreported compound **13**. The straightforward separation and isolation protocols were also able to reveal six secondary metabolites from *Parmelia aurulenta*, evernic acid **9** [16], methyl 3-hydroxy orsellinate **10** [18], methyl orsellinate **14** [12], 2,4-dihydroxy-6-pentylbenzoate **15** [12], 2-heptyl-6-hydroxy-4-methoxybenzoic acid **16** [20] and stenosporic acid **17** [21], for the first time (Figure 3).

Analysis of the ^1^H NMR spectra of the depside **5** showed four singlets assigned to the aldehyde, aromatic, methoxy and Ar-methyl protons *δ* 10.35, 6.41, 4.03, 2.59, respectively. The ^13^C NMR spectrum revealed ten resolved carbon resonances including δ 195.4, 173.4, 53.7 and 25.7, assigned to the aldehyde, carboxylic acid, methoxy and methyl C atoms, respectively (Table 1).

**Figure 4:**
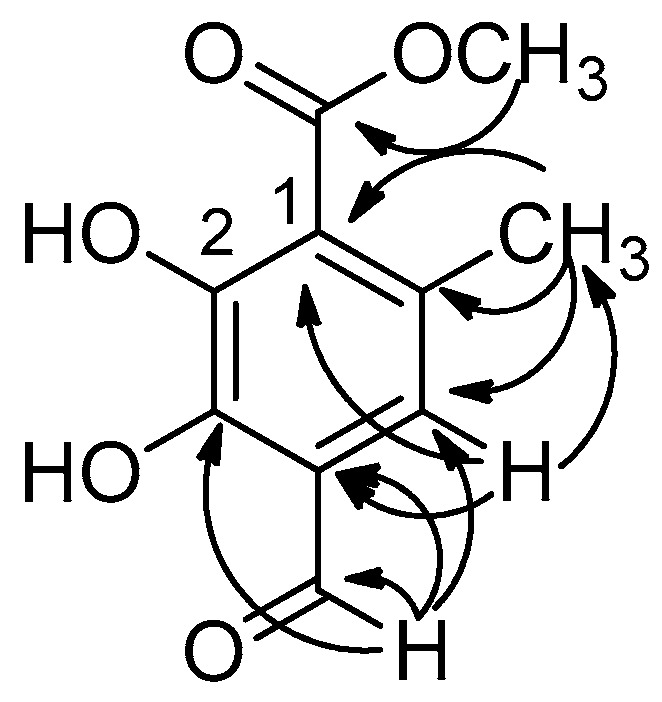
Proton–carbon correlations of methyl 4-formyl-2,3-dihydroxy-6-methylbenzoate **13**.

An HSQC spectral analysis indicated a correlation between the aldehyde resonance at *δ* 10.35 ppm to the resonance at *δ* 195.4 ppm. Further analysis found this proton to have an HMBC two-bond correlation with the resonance at *δ* 109.9 ppm assigned to C4. Analysis of the HMBC spectrum also showed the aldehyde proton correlating with resonances at *δ* 167.8 and 113.2 ppm assigned to C3 and C5, respectively (Figure 4). The resonance at *δ* 6.41 appeared as a single with one proton integration and showed an HMBC correlation with the resonances at *δ* 167.8, 106.1 and 25.6 ppm assigned to C1, C3, C6-**C**H_3_, respectively. A singlet with a 3 protons integration, *δ* 4.03 ppm was assigned to the methoxy substituent and also showed a proton–carbon correlation in the HMBC spectrum, with the resonance at *δ* 173.4 ppm. The singlet with a 3 protons integration at *δ* 2.59 ppm was assigned to C6-CH_3_ and analysis of the HMBC spectrum indicated a correlation between this methyl proton and resonances at *δ* 154.1, 113.2 and 106.2, assigned to C6, C5 and C1, respectively. Analysis of the FTIR spectrum showed a medium broad peak at 3296 and a sharp peak at 1645, and suggested the presence of hydroxyl and carbonyl functionality. Overall, the spectral analysis (Figures S1–S7 in the Supplementary Material) suggested compound **13** as methyl 4-formyl-2,3-dihydroxy-6-methylbenzoate, which was confirmed by high-resolution mass spectrometry which showed a negative molecular ion at 209.0456 *m*/*z* assigned to the molecular formula C_10_H_10_O_5_.

**Figure 5:**
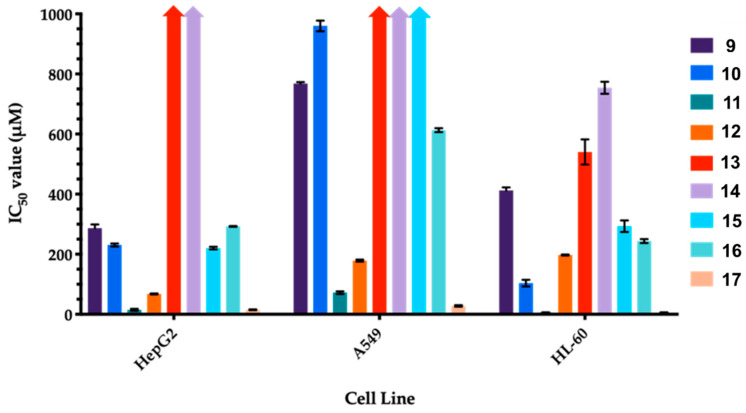
IC_50_ values obtained from the 3-(4,5-dimethylthiazol-2-yl)-2,5-diphenyltetrazolium bromide (MTT) assays of compounds 1–10 (24 h incubation) with the specified cell lines. The columns represent the mean IC_50_ value and the error bars represent the standard deviation from the mean, n = 3.

Figure 5 shows the IC_50_ values obtained for compounds **9**–**17** in the MTT assays performed with the specified cell lines. In general, the lowest overall cytotoxic (highest IC_50_ values) response was obtained from the A549 cells, whereby several compounds exhibited IC_50_ values greater than 500 µM (namely, **9**, **10**, **13**, **14**, **15** and **16**) toward the lung cancer cell line. Focussing on the individual compounds across the cell lines, it is evident that the di-depside compounds **11**, **12** and **17**consistently showed the highest cytotoxicity with IC_50_ values ranging from 6 to 180 µM (specific IC_50_ values for **11**: HepG2 cells, 15 µM; A549 cells, 72 µM; HL-60 cells, 6 µM; **12**: HepG2 cells, 68 µM; A549 cells, 180 µM; HL-60 cells, 197 µM; **17**: HepG2 cells, 15 µM; A549 cells, 28 µM; and HL-60 cells, 6 µM).

## 4. Discussion

Previous studies on the crude extract of these three lichens indicated moderate activities against several cell lines including MCF7, Widr, Hela and a normal Vero cell [13]. Evemic acid **12** was previously reported to possess anti-cancer activity against A549, DU145, MCF-7, SiHa, and U87MG with IC_50_ values of >100, 90.99, 108.29, >100, and >100 μM, respectively [17]. Methyl 3-hydroxy orsellinate **10** was previously reported with no inhibitory effect on LNCaP and DU-145 tumor cell lines using an MTT assay. Furthermore, the compound did not significantly affect the expression of proteins Bcl-2, Bax, TRAIL, COX-2, NOS2, and Hsp70 [22]. The compound **14** was previously observed to possess no cytotoxic effects against T-47D, PANC-1 and WIDR cell lines [23]. This compound was also reported to possess moderate anti-cancer activity against HEp-2 larynx carcinoma and MCF7 cell lines with an IC_50_ value > 50 µg/mL [24].

In the current study, the individual depside compounds (**9**–**17**) were evaluated for their cytotoxicity against human hepatocellular carcinoma (HepG2) cells, human lung carcinoma (A549) cells and acute promyelocytic leukemia (HL-60) cells. Importantly, the di-depsides consistently showed the greatest cytotoxicity towards all three cell lines tested, with the most promising responses observed for **11** and **17** in the acute promyelocytic leukemia cell line (HL-60 cells). Compared to didepside **12**, the lengthening of the alkyl chain (R1 = R2 = C_3_H_7_ versus R1 = R2 = CH_3_) in the molecule **11**, resulted in the greater toxicity exhibited by **11**. Similarly, the extension of R_1_ to the pentyl group didepside **17** (versus the propyl group for R1 in **12**) resulted in even greater toxicity among the di-depsides. The resultant increased lipophilicity of the molecules may increase their uptake into the cells and contribute to the observed cytotoxicity. The free acid groups on the depside derivatives are unlikely to contribute to the anticancer activity based on a comparison against the esterified containing depsides. The C3 methyl group on depside **9** did not invoke a distinct activity whereas the different alkyl groups at C6 of the depsides derivatives did influence cytotoxicity. This pattern has been previously shown with the didepsides (**11**, **12** and **17**) derivatives in this study.

This suggests that it would be valuable to perform future studies to determine the mode of action of these compounds and their molecular target(s) in the cancer cells. This would create a starting point for the design of synthetic derivatives with enhanced toxicity towards the cancer cells.

## 5. Conclusions

The phytochemical studies successfully isolated nine depsides, which included six mono-depsides and three di-depsides (**9**–**17**), including the previously unreported depside methyl 4-formyl-2,3-dihydroxy-6-methylbenzoate **13** from three Indonesian folious lichen. Cytotoxicity evaluations of the isolates against HepG2, A549 and HL-60 cell lines revealed consistent activity of the didepsides (**11**, **12**, **17**) in which it is believed that the lipophilic alkyl chains contributed to their comparative activity whereby **17** exhibited the highest biological activity.

## Figures and Tables

**Table 1:** ^1^H and ^13^C NMR spectroscopic data for compound **13** (in acetone-*d*_6_).

	13	
	*δ*_H_ (*J* in Hz)	*δ*_C_
C(=O)OH		173.4
1		106.2
2		169.2
3		167.9
4		109.9
5	6.41, s	113.2
6		154.2
C(=O)H	10.35-COH	195.4
CH_3_	2.59, s	25.7
OCH_3_	4.03, s	53.7

## Supplementary Materials

The following are available online at https://www.mdpi.com/2218-273X/10/10/1420/s1, Figure S1: ^1^H-NMR spectrum of compound **13** in acetone-*d*_6_, Figure S2: ^13^C-NMR spectrum of compound **13** in acetone-*d*_6_, Figure S3: gCOSY-NMR spectrum of compound **13** in acetone-*d*_6_, Figure S4: gHSQC-NMR spectrum of compound **13** in acetone-*d*_6_, Figure S5: gHMBC-NMR spectrum of compound **13** in acetone-*d*_6_, Figure S6: IR spectrum of compound **13**, Figure S7: HRESI spectrum of compound **13**, Figure S8: Chromatogram of preparative HPLC of *Pysicia millegrana* (top), *Parmelia dilatata* (Middle) and *Parmelia aurulenta* (bottom), Figure S9: Concentration–response curves for the MTT assay of the specified compounds tested against HepG2 cells. The data points represent the mean of 6 points and the error bars represent the standard deviation from the mean. Figure S10: Concentration–response curves for the MTT assay of the specified compounds tested against A549 cells. The data points represent the mean of 6 points and the error bars represent the standard deviation from the mean. Figure S11: Concentration-response curves for the MTT assay of the specified compounds tested against HL-60 cells. The data points represent the mean of 6 points and the error bars represent the standard deviation from the mean.
