# Supplementary material for: Anti-cancer Evaluation of Depsides Isolated from Indonesian Folious Lichens: Physcia millegrana, Parmelia dilatata and Parmelia aurulenta
Source: Biomolecules. 2020 Oct 8;10(10):1420. doi: 10.3390/biom10101420 (PMC7600581; doi:10.3390/biom10101420)
Supplement: Supplementary file 1 [file biomolecules-10-01420-s001.pdf]

biomolecules

Supplementary Materials

Ari Satia Nugraha [1,*], Tinton Agung Laksono [1], Lilla Nur Firli [1], Chintya Permata Zahky Sukrisno Putri [1], Dwi Koko Pratoko [1], Zulfikar Zulfikar [1], Ludmilla Fitri Untari [2], Hendris Wongso [3,4], Jacob M. Lambert [3], Carolyn T. Dillon [3] and Paul A. Keller [3,*]

[1] Drug Utilisation and Discovery Research Group, Faculty of Pharmacy, University of Jember, Jember, Indonesia 68121; tintona.l@gmail.com (T.A.L.); lillanurfirli13@gmail.com (L.N.F.); chintyapermata97@gmail.com (C.P.Z.S.P.); dwikoko.farmasi@unej.ac.id (D.K.P.); zulfikar.nazar@gmail.com (Z.Z.)

[2] School of Biology, Faculty of Biology, Gadjah Mada University, Yogyakarta, Indonesia 55281; ludmilla.untari@ugm.ac.id

[3] School of Chemistry & Molecular Bioscience and Molecular Horizons, University of Wollongong, and Illawarra Health & Medical Research Institute, Wollongong, NSW, 2522, Australia; hw765@uowmail.edu.au (H.W.); jl247@uowmail.edu.au (J.M.L.); carolynd@uow.edu.au (C.T.D.)

[4] Labelled Compound and Radiometry Division, Center for Applied Nuclear Science and Technology, National Nuclear Energy Agency, Bandung 40132, Indonesia

* Correspondence: arisatia@unej.ac.id (A.S.N.); keller@uow.edu.au (P.A.K.); Tel.: +62-3-3132-4736 (A.S.N.); +61-2-4221-4692 (P.A.K.)

Received: 30 August 2020; Accepted: 22 September 2020; Published: date

Abstract: Cancer is a serious health burden on global societies. The discovery and development of new anti-cancer therapies remains a challenging objective. Although it has been shown that lichen secondary metabolites may be potent sources for new anti-cancer agents, the Indonesian- grown folious lichens, *Physcia millegrana, Parmelia dilatata* and *Parmeila aurulenta,* have not yet been explored. In this study exhaustive preparative high-performance liquid chromatography was employed to isolate the lichen constituents with spectroscopic and spectrometric protocols identifying nine depsides **9–17**, including the new methyl 4-formyl-2,3-dihydroxy-6-methylbenzoate **13**. The cytotoxicity of the depsides towards cancer cells was assessed using the 3-(4,5-dimethylthiazol-2-yl)-2,5-diphenyltetrazolium bromide (MTT) assay. The results indicated lowest toxicity of the depsides towards human A549 lung cancer cells. Importantly, the di-depsides (**11**, **12** and **17**) showed greatest toxicity, indicating that these structures are biologically more active than the mono-depsides against the HepG2 liver cancer, A549 lung cancer and HL-60 leukemia cell lines.

Keywords: Indonesia; lichen; *Physcia millegrana*; *Parmelia dilatata*; *Parmelia aurulenta*; depsides; anti-cancer

---

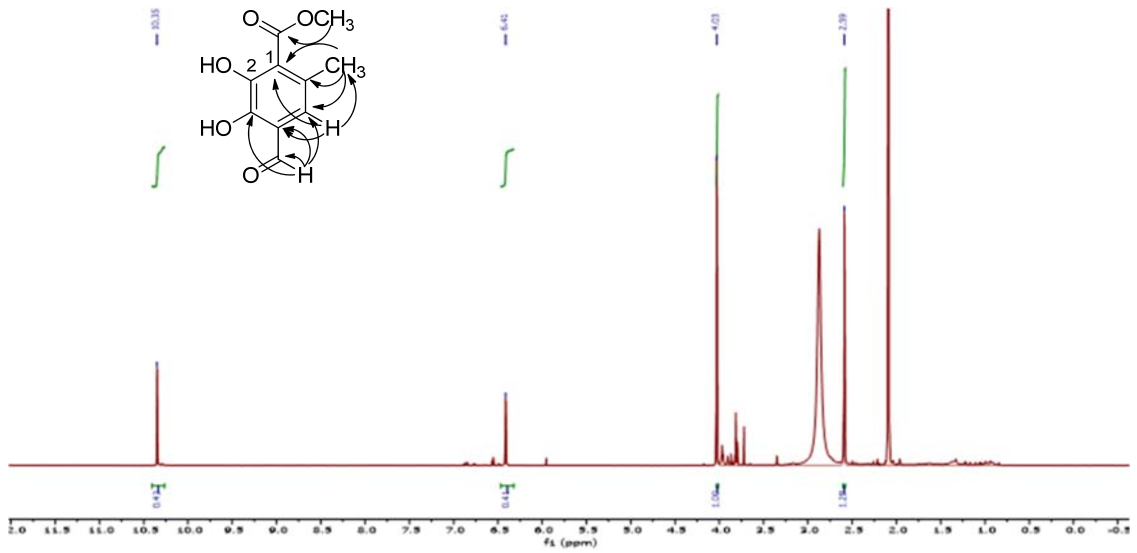

Figure S1. ^{1}H-NMR spectrum of compound **13** in acetone-*d6*.

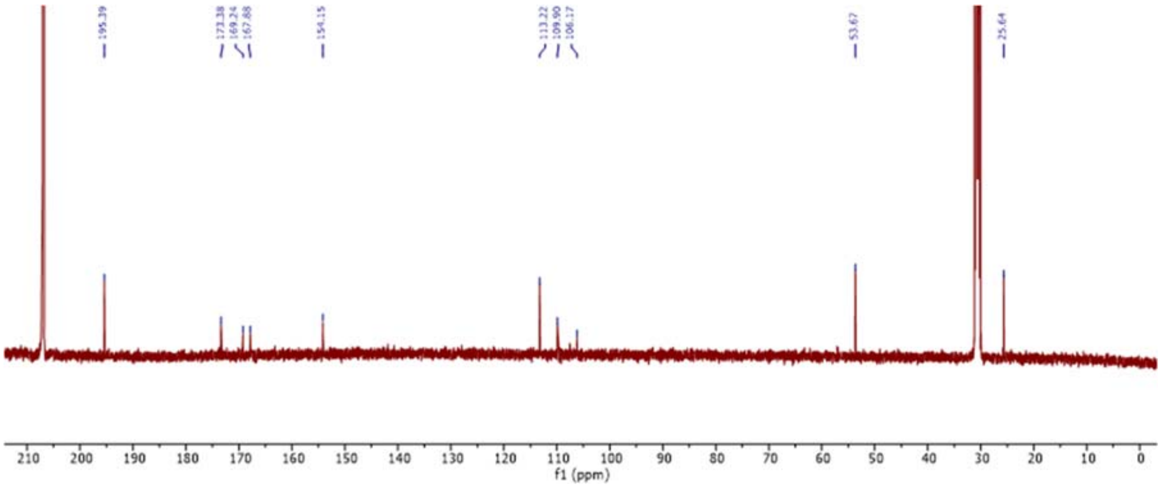

Figure S2. ^{13}C-NMR spectrum of compound **13** in acetone-*d6*.

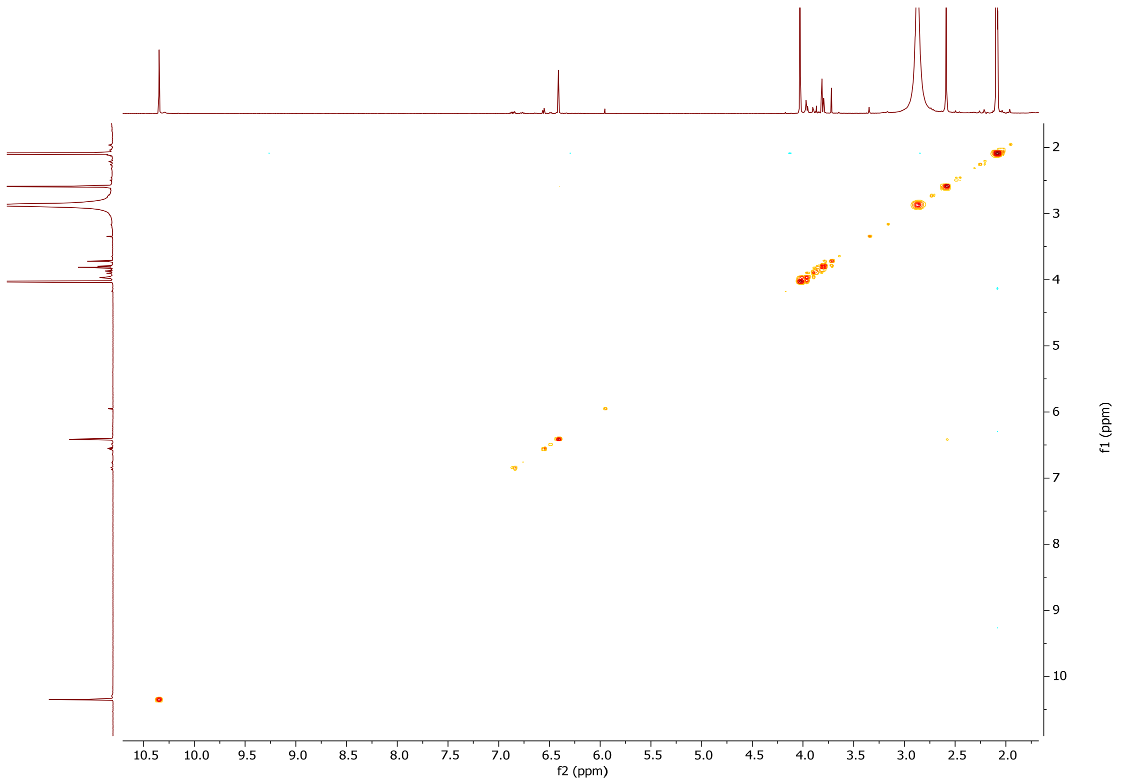

Figure S3. gCOSY-NMR spectrum of compound **13** in acetone-*d6*.

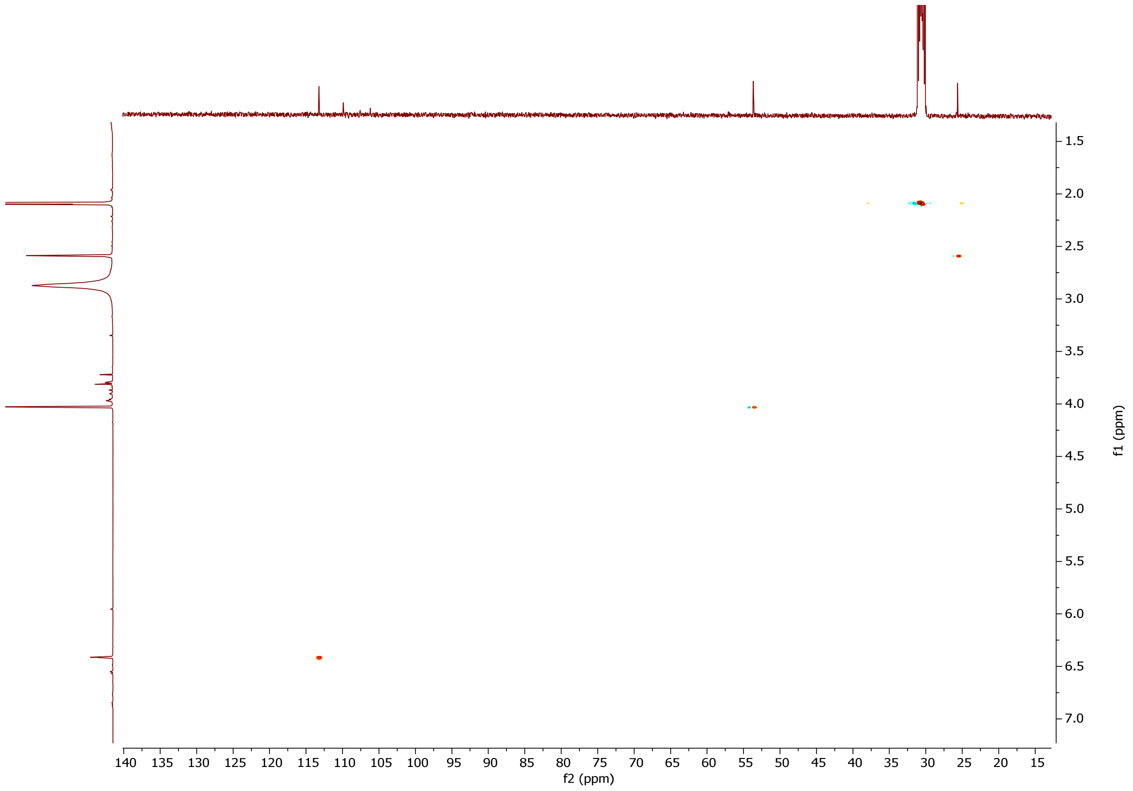

Figure S4. gHSQC-NMR spectrum of compound **13** in acetone-*d6*.

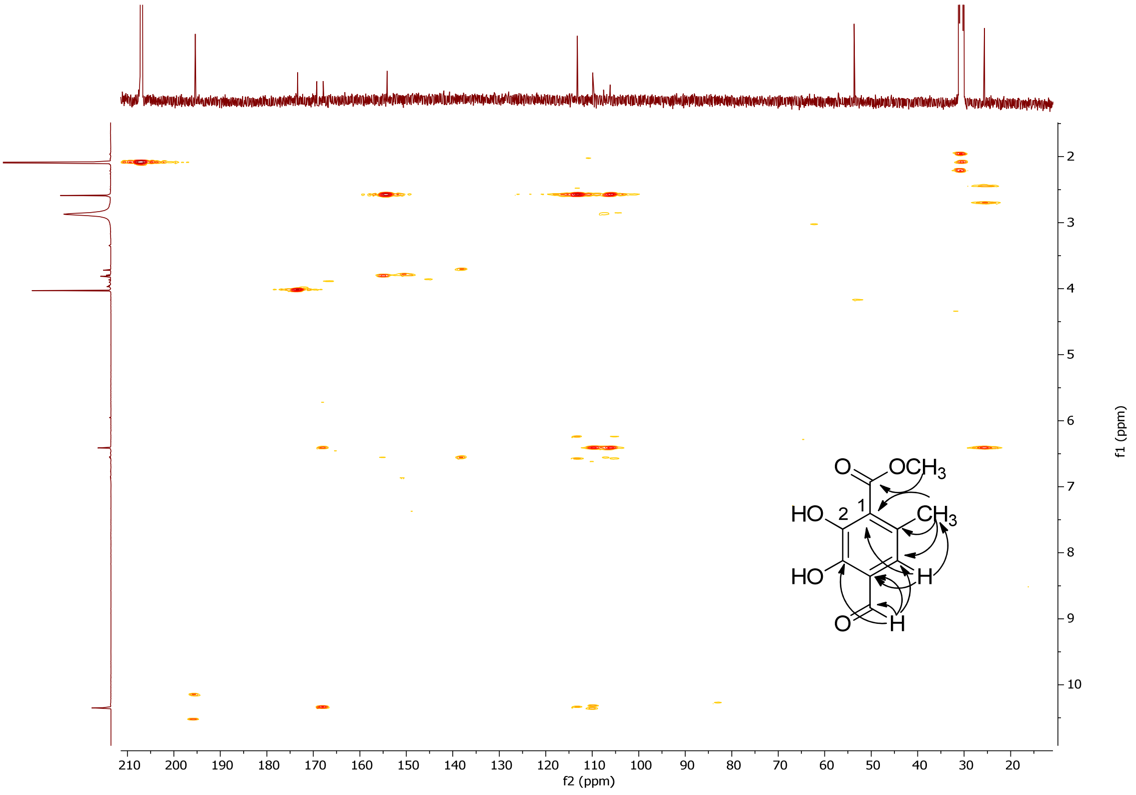

Figure 5. gHMBC-NMR spectrum of compound **13** in acetone-*d6*.

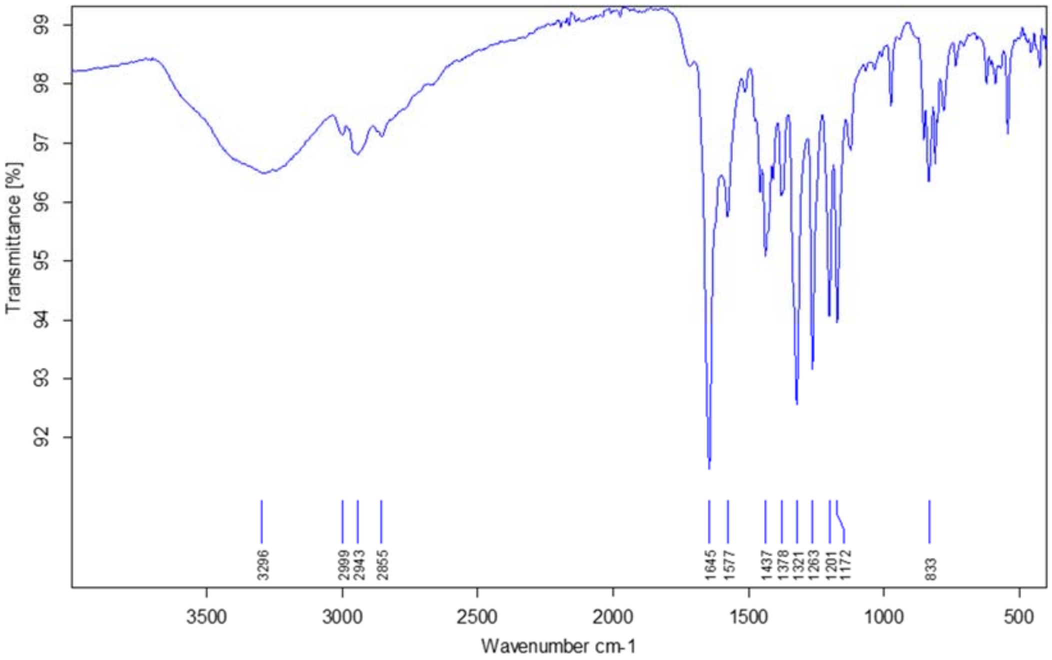

Figure S6. IR spectrum of compound **13.**

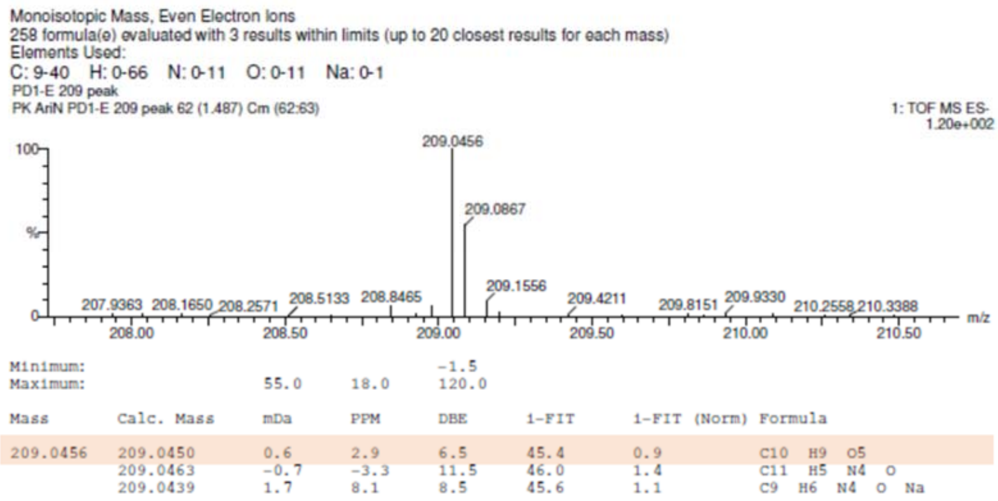

Figure S7. HRESI spectrum of compound **13.**

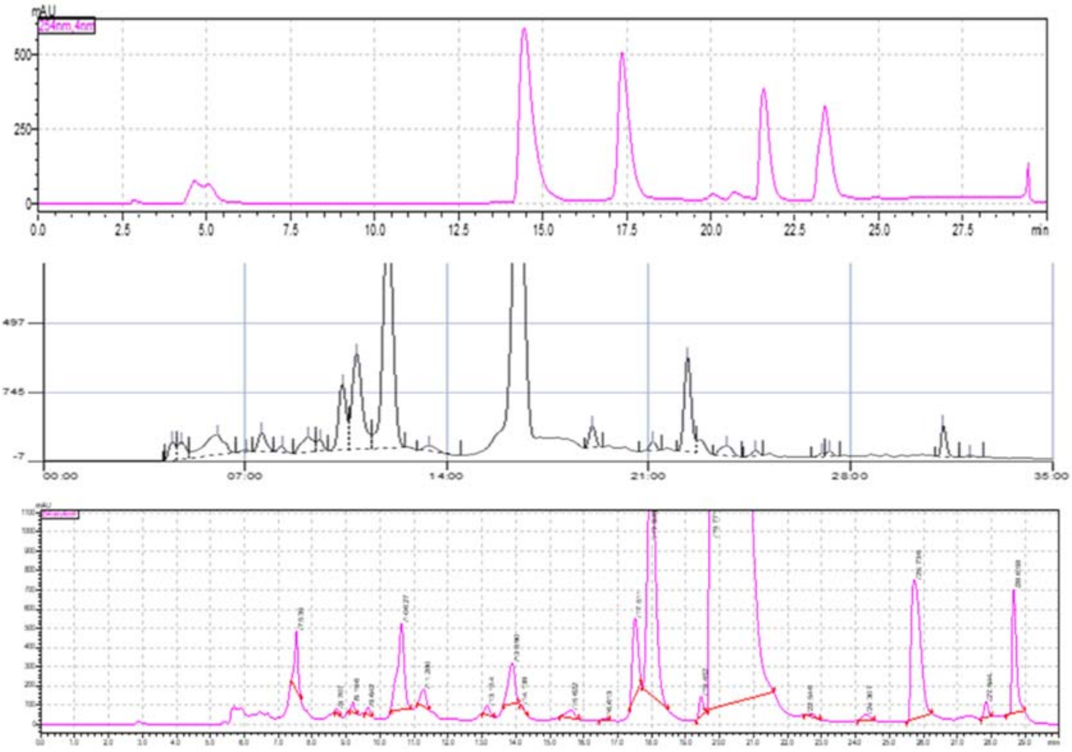

Figure S8. Chromatogram of preparative HPLC of *Pysicia millegrana* (top), *Parmelia dilatata* (middle) and *Parmelia aurulenta* (bottom).

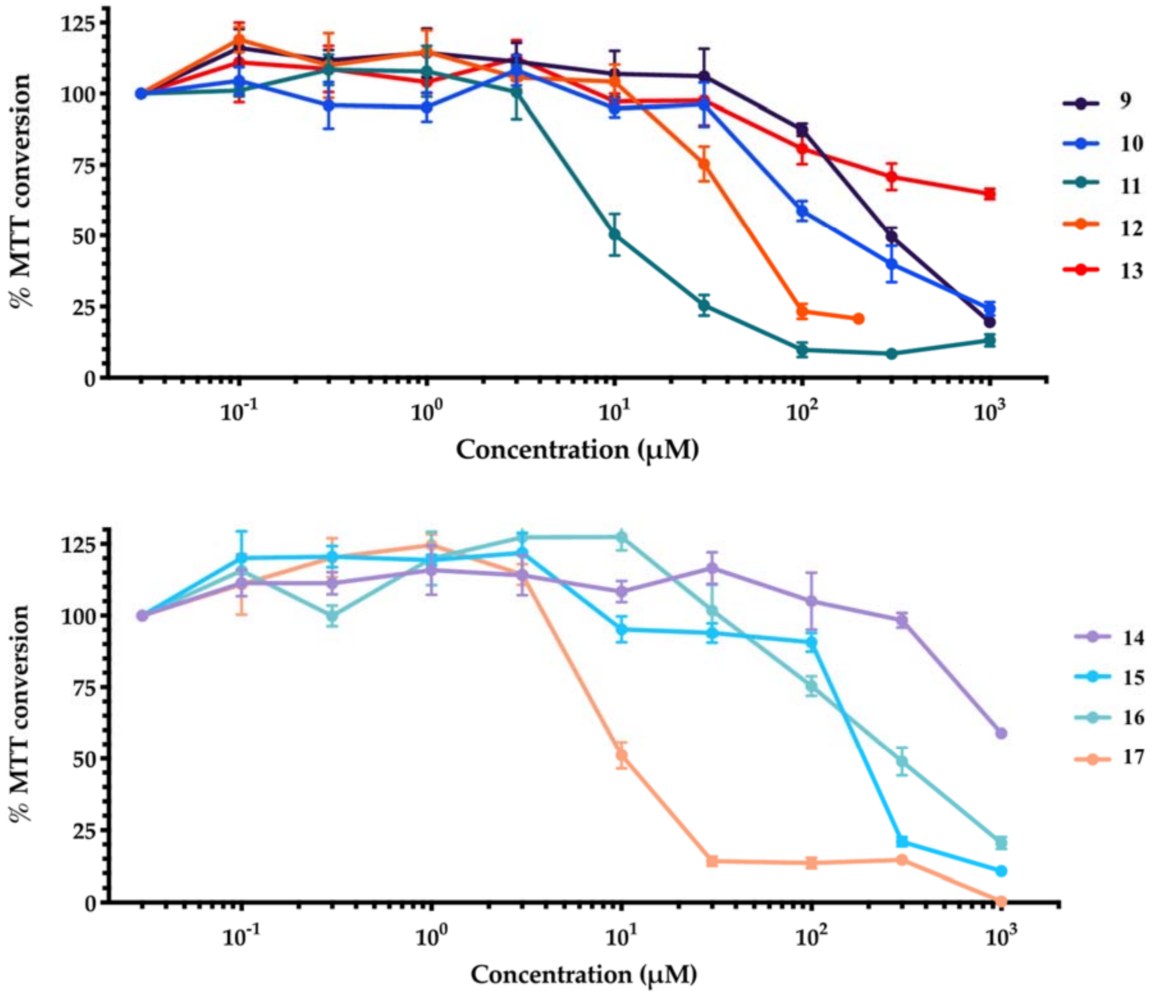

Figure S9. Concentration-response curves for the MTT assay of the specified compounds tested against HepG2 cells. The data points represent the mean of 6 points and the error bars represent the standard deviation from the mean.

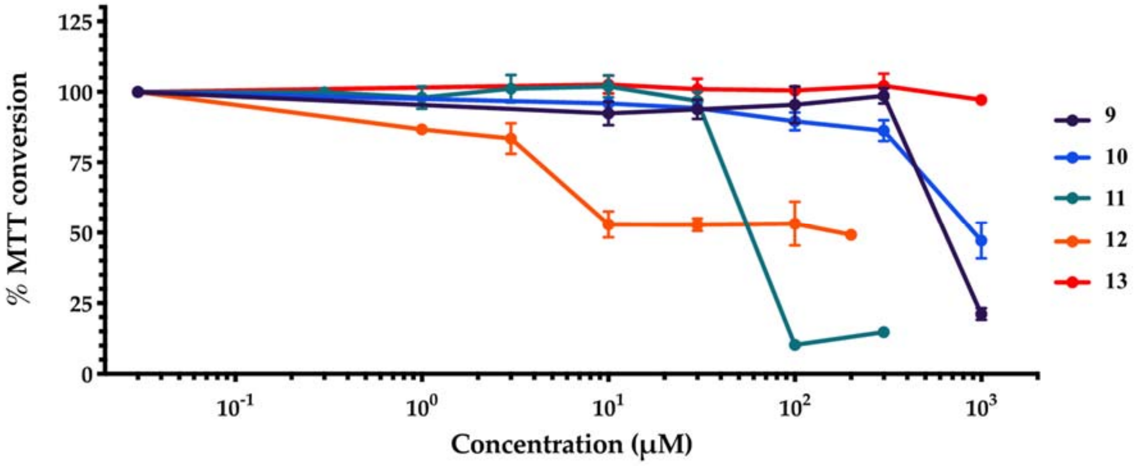

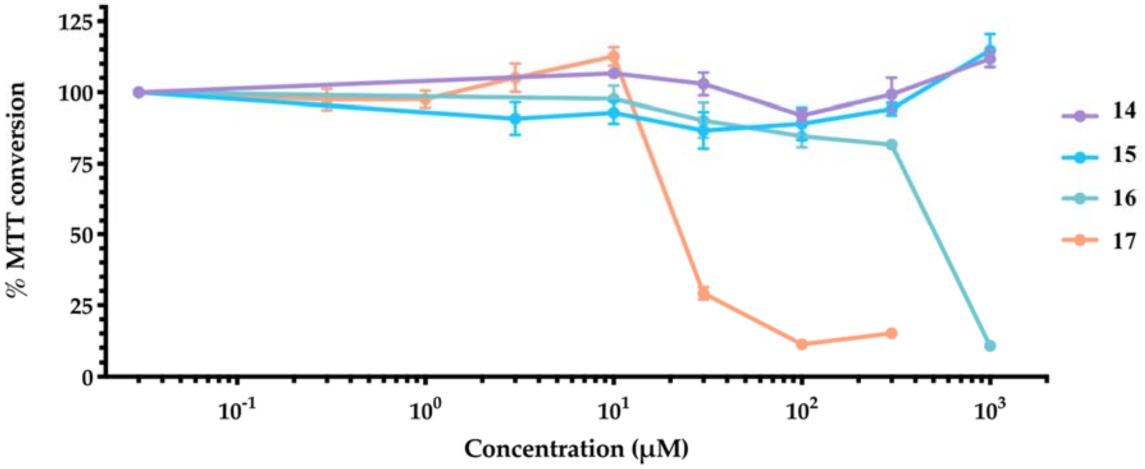

Figure S10. Concentration-response curves for the MTT assay of the specified compounds tested against A549 cells. The data points represent the mean of 6 points and the error bars represent the standard deviation from the mean.

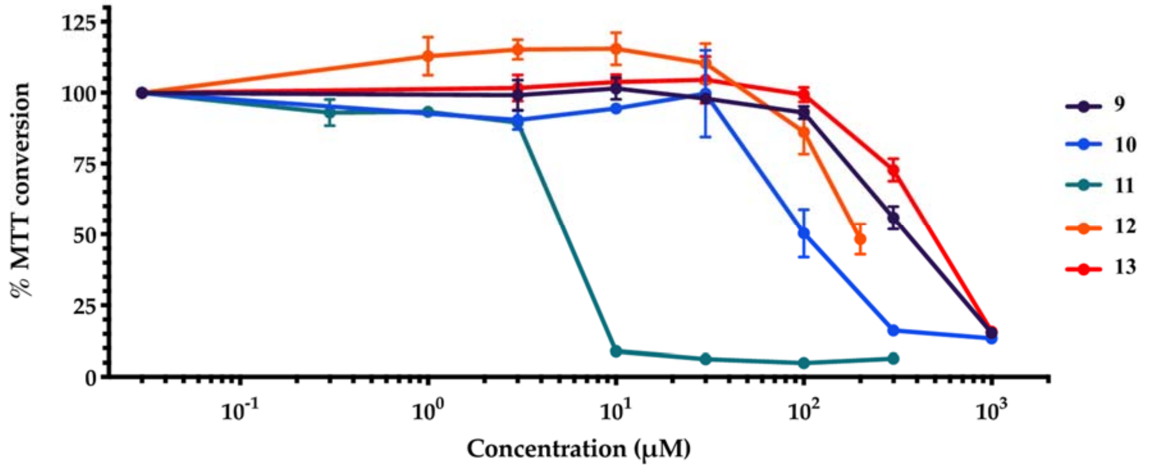

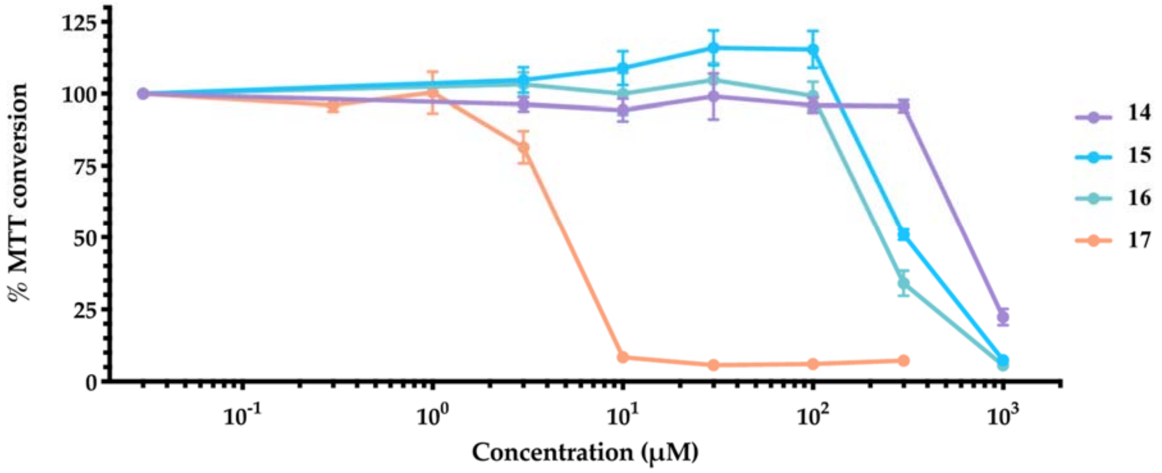

Figure S11. Concentration-response curves for the MTT assay of the specified compounds tested against HL60 cells. The data points represent the mean of 6 points and the error bars represent the standard deviation from the mean.

© 2020 by the authors. Submitted for possible open access publication under the terms and conditions of the Creative Commons Attribution (CC BY) license (http://creativecommons.org/licenses/by/4.0/).
